# New *Life* is coming: committed to improving human health

**DOI:** 10.1093/lifemedi/lnac001

**Published:** 2022-06-14

**Authors:** Guang-Hui Liu, Juan Carlos Izpisua Belmonte

**Affiliations:** Beijing Institute for Stem Cell and Regenerative Medicine, Beijing 100101, China; Institute for Stem Cell and Regeneration, Chinese Academy of Sciences, Beijing 100101, China; State Key Laboratory of Membrane Biology, Institute of Zoology, Chinese Academy of Sciences, Beijing 100101, China; Advanced Innovation Center for Human Brain Protection, National Clinical Research Center for Geriatric Disorders, Xuanwu Hospital Capital Medical University, Beijing 100053, China; Gene Expression Laboratory, Salk Institute for Biological Studies, La Jolla, CA 92037, USA

According to the World Population Prospects report released by the United Nations Department of Economic and Social Affairs in 2019, 1 in 11 people was over 65 years old globally and the percentage was predicted to continue to rise to one in every six people by around 2050. Aging, a process of functional decline of the body that occurs with the passing of time, is one of the biggest risk factors for multiple chronic diseases, including neurodegenerative diseases, cardiovascular diseases, diabetes, and cancer. With the increasing average age of the global population, aging and its associated health conditions have become common health challenges around the globe. Individuals with chronic diseases can typically maintain certain degrees of quality of life through standard medical care, but they are often highly vulnerable when facing large-scale infectious diseases. For instance, most victims of COVID-19, which has affected more than 500 million individuals and caused more than 6 million deaths worldwide so far, are elderly or individuals with chronic diseases. The aging of the global population and the increasing number of individuals with diseases unveil the necessity of focusing our attention on disease prevention and treatment. With this in mind and with the goal of helping to solve global health challenges, we are pleased to launch *Life Medicine*.

*Life Medicine* is a bimonthly journal focusing on basic and translational medical research published by Higher Education Press and Oxford University Press and is one of the flagship journals in the *Life* series. The mission of *Life Medicine* is to accelerate the translation of research from the bench to the bedside by providing a platform for the exchange of cutting-edge knowledge and fresh ideas among biomedical researchers and clinicians.

*Life Medicine* publishes various types of papers with strong readability, including Articles, Resources, Reviews, Letters, Previews, Editorials, Research Highlights, News & Opinions, and other contents commissioned from scientists in the field of biomedical research to promptly disseminate latest significant advances in basic and translational, and clinical medicine. With the goal to continuously provide understanding on disease mechanisms and improve human health, *Life Medicine* seeks to publish high-quality and high-impact research work in fields including, but not limited to, health science, intelligent medicine, reproductive medicine, geriatric medicine, regenerative medicine, pharmacology, genetic diseases, infectious diseases, cardiovascular diseases, respiratory diseases, digestive diseases, neurological diseases, and immunological disorders.

The current editorial board of *Life Medicine* includes internationally renowned biological and biomedical researchers with extensive research experience and a global vision covering various fields of basic and translational medicine from many countries.

We truly appreciate every effort in the understanding and implementation of disease diagnosis, prevention, and treatment as well as improving the quality of life to support human well-being. We sincerely look forward to your attention and active communication. Please check the latest information of *Life Medicine* through our official website (https://academic.oup.com/lifemedi), Twitter account (@LifeMedJournal), and WeChat account (Life Medicine).

